# Towards implementation of AI in New Zealand national diabetic screening program: Cloud-based, robust, and bespoke

**DOI:** 10.1371/journal.pone.0225015

**Published:** 2020-04-10

**Authors:** Li Xie, Song Yang, David Squirrell, Ehsan Vaghefi

**Affiliations:** 1 School of Optometry and Vision Sciences, The University of Auckland, Auckland, New Zealand; 2 School of Computer Sciences, The University of Auckland, Auckland, New Zealand; 3 Department of Ophthalmology, The University of Auckland, Auckland, New Zealand; 4 Auckland District Health Board, Auckland, New Zealand; 5 Auckland Bioengineering Institute, The University of Auckland, Auckland, New Zealand; Universita degli Studi di Firenze, ITALY

## Abstract

Convolutional Neural Networks (CNNs) have become a prominent method of AI implementation in medical classification tasks. Grading Diabetic Retinopathy (DR) has been at the forefront of the development of AI for ophthalmology. However, major obstacles remain in the generalization of these CNNs onto real-world DR screening programs. We believe these difficulties are due to use of 1) small training datasets (<5,000 images), 2) private and ‘curated’ repositories, 3) locally implemented CNN implementation methods, while 4) relying on measured Area Under the Curve (AUC) as the sole measure of CNN performance. To address these issues, the public EyePACS Kaggle Diabetic Retinopathy dataset was uploaded onto Microsoft Azure^™^ cloud platform. Two CNNs were trained; 1 a “Quality Assurance”, and 2. a “Classifier”. The Diabetic Retinopathy classifier CNN (DRCNN) performance was then tested both on ‘un-curated’ as well as the ‘curated’ test set created by the “Quality Assessment” CNN model. Finally, the sensitivity of the DRCNNs was boosted using two post-training techniques. Our DRCNN proved to be robust, as its performance was similar on ‘curated’ and ‘un-curated’ test sets. The implementation of ‘cascading thresholds’ and ‘max margin’ techniques led to significant improvements in the DRCNN’s sensitivity, while also enhancing the specificity of other grades.

## Introduction

It is estimated that by 2040, nearly 600 million people will have diabetes worldwide [1]. Diabetic retinopathy (DR) is a common diabetes-related microvascular complication, and is the leading cause of preventable blindness in people of working age worldwide [2, 3]. It has been estimated that the overall prevalence of non-vision-threatening DR, vision-threatening DR and the blinding diabetic eye disease were 34·6%, 10·2%, and 6·8% respectively [3–6]. Clinical trials have shown that the risk of DR progression can be significantly reduced by controlling major risk factors such as hyperglycaemia and hypertension [7–9]. It is further estimated that screening, appropriate referral and treatment can reduce the vision loss from DR by 50% [10–12]. However, DR screening programs are expensive to implement and administer and even in developed countries it is estimated that these programs do not reach up to 30% of people with diabetes [13, 14]. Whilst the reasons patients do not present for eye screening vary, the lack of a readily accessible local screening site or associated cost in absence of a public screening service, are significant barriers to many.

Artificial intelligence (AI) and its subcategory of Deep Learning have gained popularity in medical images processing, including DR screening. In deep learning, a convolutional neural network (CNN) is designed and trained based on large datasets of ground truth data and disease labels. The CNN algorithm adjusts its weights and discovers which features to extract from medical data (e.g. fundus photos) to achieve the best classification accuracy, when compared to human performance [15–20], with one study (De Fauw et al.) demonstrating that its AI was 2%-5% more accurate than 6 out of the 8 experienced clinicians in detecting referable-DR. CNNs use layers with convolutions, which are defined as mathematical functions that use filters to extract features from an image [21–23]. The output of a DR classifying CNN can be either a binary classification such as Healthy vs Diseased; or a multi-class classification task such as Healthy, Non-referable DR, Referable DR [16, 24].

The rapid initial advances of AI, especially in DR classification, raised expectations that AI would be rapidly implemented within national DR screening programs, with attendant and noticeable cost savings [25, 26]. To date, these systems have yet to be successfully translated into clinical care, in a large part due to major generalizability issues of research-built AIs. Some of the major flaws of research-built AIs that are hindering their generalizability are: 1) using small training (<5,000 images) datasets, 2) repositories that are often private and ‘curated’ to remove images that are deemed to be of low quality, and 3) lack of external validation [17, 27–29]. Although 5,000 images for a training dataset is an arbitrary number, it has been shown that large training datasets lead to improved performance and generalizability [30, 31]. These issues are often observed in research-driven AIs and have led to a slew of extremely biased DR classifying neural networks in the published literature. Some recent publications have pointed out the lack of generalizability of even the best of these AIs [32–35].

Our extensive investigation (to be published soon as a systematic review) has found only a few published research-based AIs that could be closer to clinical translation [4, 16, 18, 19, 26, 36–38]. Although admirable achievements in their own right, these AIs often need dedicated and TensorFlow compatible graphic cards (GPUs) to achieve rapid live image grading and in reality public health providers rely on older and\or less expensive IT infrastructure, which means that such a high computational demand would hinder their clinical translation.

Finally, the creators of DR-screening AIs have traditionally focused on improving the accuracy of their trained AIs, as measured by the AUC [17]. Although reasonable, it should be noted that different diabetic eye screening programs will have different requirements. Established programs, such the public screening system in New Zealand [39, 40] are designed to delineate those patients with no/low risk disease from those with high risk “sight threatening” disease. In this scenario a classifier CNN which is highly sensitive to sight threatening disease and has a very high negative predictive value for a negative result, will potentially remove the need for a significant (>80%) portion of images to be sent for human review, with an immediate and significant cost saving for the program. However, in rural community-based screening programs, operating in remote and/or low socioeconomic regions on portable handheld cameras where it is also important to identify those patients who would also benefit from review of their systemic disease, arguably the emphasis should be on delineating those patients with no disease from those with any disease in order to identify those individuals who need further review. In such cases a high positive predictive value for a normal result may be more appropriate. There are then opportunities to build bespoke DRCNN’s tailored to the needs of individual screening programs.

In this, paper we report the use of two established post training techniques; cascading thresholds and margin max to manipulate the sensitivity and specificity of a bespoke DRCNN towards: 1. No disease and 2. Sight threatening DR. We are actively pursuing clinical implementation of our AIs and as such our recent findings would be of great interest for similar groups around the world.

## Methodology

**Fig 1:**
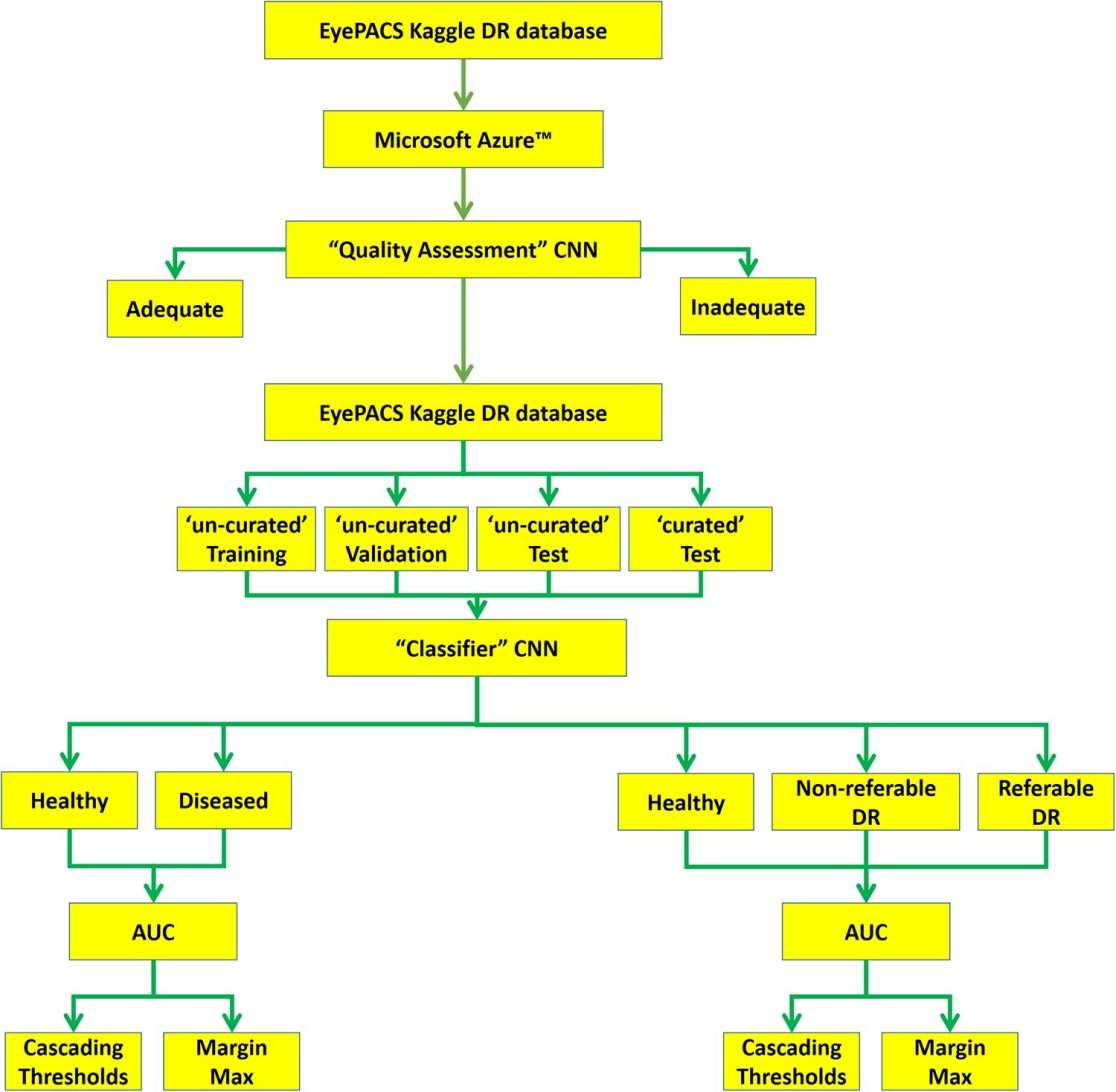
Flowchart of our AI design, implementation and test. The public data attaiment and upload onto the Microsoft Azure cloud platform was the first step. “quality assessment” CNN was trained to identify adequate and inadequate images. the entire public dataset was then devided to training, validation and test sets. The test set was then ‘curated’ by the “quality assessment” CNN. The DRCNN was trained on un-curated data, and then tested on ‘curated’ and ‘un-curated’ data. Its performance was also assessed using 2 or 3 DR labels.

Here is a short summary of the methodological steps in this project, which are explained in more details subsequently. The original EyePACS Kaggle DR dataset was obtained and uploaded onto the Microsoft Azure^™^ platform. Initially, a “Quality Assessment” CNN was trained for assessing the quality of the retinal images. Next and to better match the New Zealand grading scheme, the original grading was modified in two ways [Healthy vs Diseased] and [Healthy vs Non-referable DR vs Referable DR]. The uploaded dataset was then divided into training (70%), validation (15%) and test (15%) sets. A separate DR classifier CNN was then trained on the Microsoft Azure^™^ platform, using the ‘un-curated’ training and validation datasets. The not-seen-before test set was then analysed by the “Quality Assessment” CNN thus creating a ‘curated’ test set in addition to the original ‘un-curated’ set. The performance of the DRCNN was then assessed using both ‘curated’ and ‘un-curated’ test sets. Finally, the ‘cascading threshold’ and ‘margin max’ techniques were implemented post-training, to investigate their effects on boosting the sensitivity of the DRCNN [Fig 1].

### Quality assessment dataset

**Fig 2:**
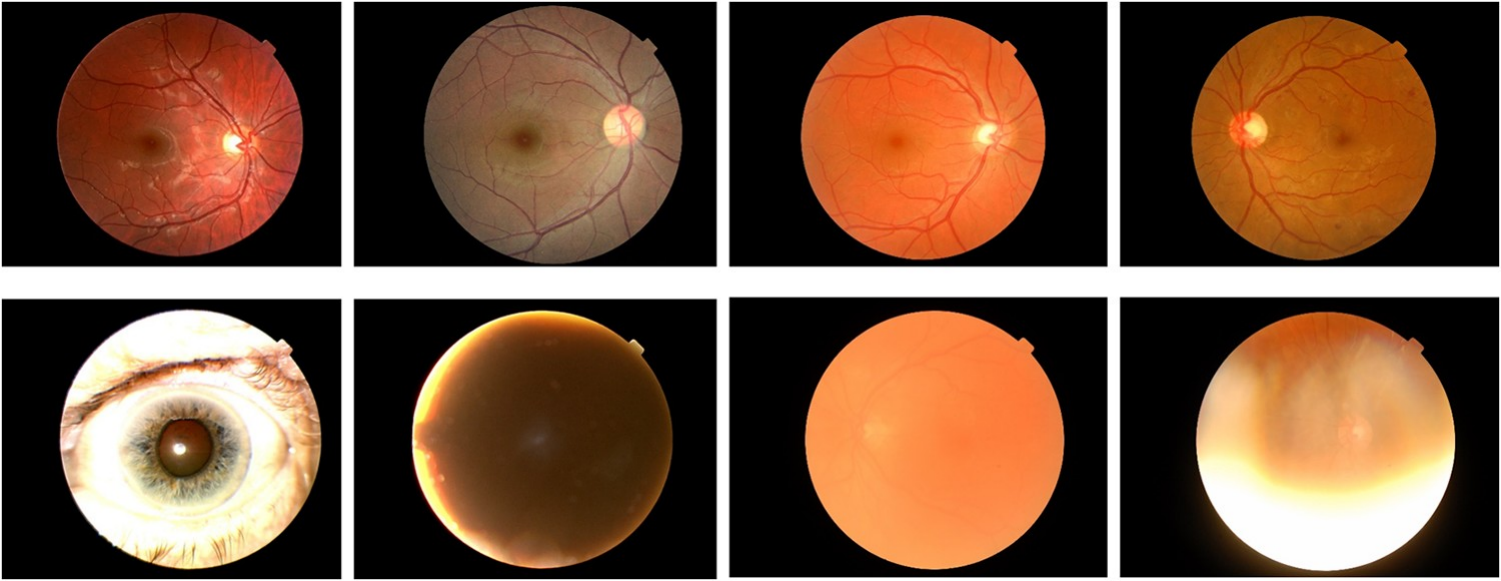
Samples of ‘adequate’ and ‘in-adequate’ images as decided by a senior retinal specialist. Fundus images deemed adequate are shown in the upper row. Fundus images deemed inadequate are shown in the bottom row.

A subset of 7,026 number of images (8% of the entire dataset), randomly chosen from the original set were used for creating the “Quality Assessment” CNN. The images were audited by a senior retinal specialist (DS) and labelled as ‘adequate’ or ‘inadequate’ (3400 \ 3626) respectively [Fig 2]. They were then split into (70%) training, (15%) validation and (15%) testing sets.

### “Quality-assessment” CNN architecture

To choose the optimum CNN design, several architectures were tested on Microsoft Azure^™^ cloud platform. These included ResNet, DenseNet, Inception and Inception-ResNet and Inception-ResNet-V2 [41]. The “Quality-Assessment” CNN was then based on a modified version of the InceptionResNet-V2 architecture. This particular neural network structure, in our experience, has faster convergence and avoids converging onto local minima. For our purposes, the number of neurons in the final output layer was changed to two, corresponding to ‘adequate’ and ‘inadequate’ classes. The learning rate was 0.001, using ADAM optimizer, with a mini-batch size of 30, and training was continued to 140 epochs.

### Classifier dataset

The public Kaggle Diabetic Retinopathy was downloaded through EyePACS, which can be found in https://www.kaggle.com/c/diabetic-retinopathy-detection/data. This dataset contains 88,700 high-resolution fundus images of the retina, labelled as No DR, Mild, Moderate, Severe, Proliferative DR. To mimic the decision making of the New Zealand national DR Screening program, the original grading was remapped to three cohorts of Healthy, Non-referable DR and Referable DR [Table 1]. Furthermore, as one potential gain of using an AI in DR Screening program is to quickly identify those that are healthy, separately the dataset was remapped to the broad classification of Healthy vs Diseased [Table 2].

**Table 1:** Re-categorization of the original Kaggle EyePACS grading scheme (5 grades) to three new categories.

Original Grade	New Grade	Number of images
No DR	Healthy	65,300
Mild DR	Non-referable DR	19,400
Moderate DR		
Severe DR	Referable DR	4,000
Proliferative DR		

**Table 2:** Re-categorization of the original Kaggle EyePACS grading scheme (5 grades) to two new categories.

Original Grade	New Grade	Number of images
No DR	Healthy	65,300
Mild DR	Diseased	23,400
Moderate DR		
Severe DR		
Proliferative DR		

Each re-categorized dataset was then split into training set, validation set and testing set with corresponding ratios of 70%, 15% and 15% respectively. The ‘curated’ dataset was created by excluding lower quality images from the ‘un-curated’ set, as identified by the “Quality Assessment” CNN.

### Pre-processing

The Kaggle EyePACS images were cropped and resized to 600*600. The choice of image size was to minimize the computational load on the Microsoft Azure^™^ platform, while not compromising the performance of the trained CNNs. According to existing literature [42] and based on our experience, larger image sizes would have led to diminishing returns in accuracy and overall performance of the designed CNNs. The resized cropped images were enhanced by applying a Gaussian blur technique [43], using the equation below.

$$I_{c}=\alpha I+\beta G(\rho )*I+\gamma$$

A series of Gaussian blur parameters were tried and an optimum set was chosen by a senior retinal specialist (DS):

α=3,β=-3,γ=128,ρ=14

**Fig 3:**
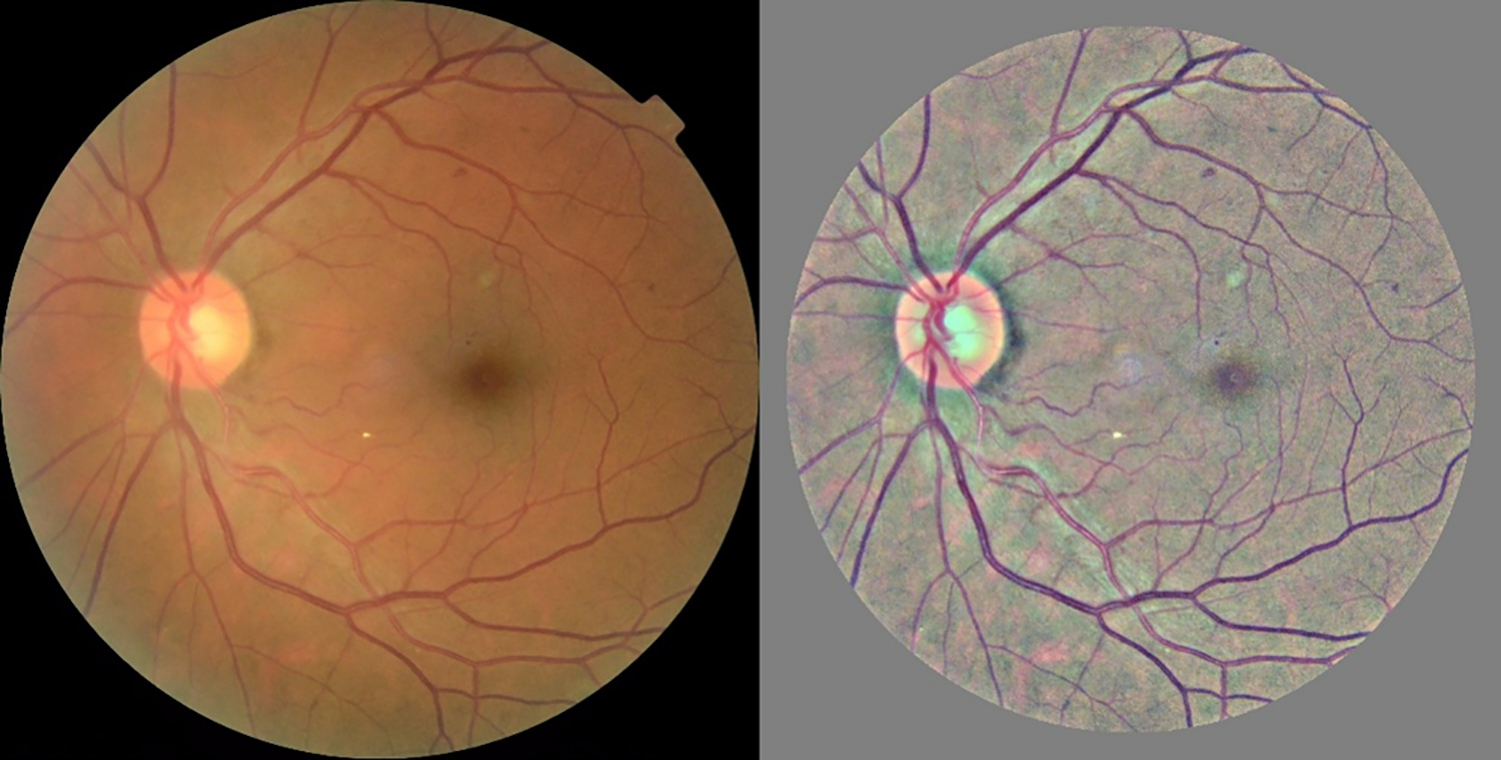
Contrast enhancement of the Kaggle EyePACS fundus image. The Gaussian blur technique was applied to the raw fundus image (left). This technique minimizes intensity and contrast variability in fundus image dataset (right).

The Gaussian blur technique has been designed to remove the variation between images due to differing lighting conditions, camera resolution and image qualities [Fig 3].

### DRCNN architecture

The DRCNN was then designed based on the Inception-ResNet-V2 architecture, since this architecture has outstanding network capacity, faster convergence speed and better stability, which are critical when training utilizing such a large dataset. Three sequential layers of a GlobalAveragePooling2D layer, a dropout layer (dropout rate = 0.3) and a fully connected layer were added to the original architecture. The activation function of the added dense layer was a Softmax function; and cross-entropy loss/error was utilized as the loss function, while Adam algorithm was utilized as the optimizer. The CNN was initially trained using the ImageNet dataset (i.e. transfer learning). An image shuffling function was applied prior to each mini-batch assembly and mini-batches were normalized prior to each epoch. In machine learning literature, k-fold cross validation is routinely used for detect any potential model’s bias. Here, due to the size of the dataset and the cloud implementation of DRCNN (i.e. resource constraints), it was ensured that the DRCNN is unbiased in its performance by using randomly-split training, validation and testing sets.

**Fig 4:**
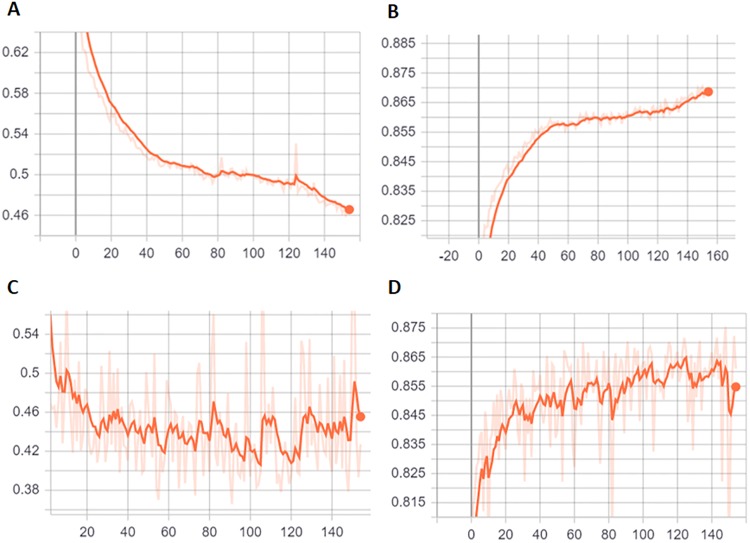
Training and validation process of DRCNN, with training cross-entropy loss (A), training accuracy (B), validation cross-entropy loss (C) and validation accuracy (D) are presented.

The learning rate was 0.001 and a mini-batch size of 64 was used for model training, and training was continued for 140 epochs [Fig 4]. Finally, a weighted loss-function was used here to address the class imbalance of the Kaggle EyePACS dataset. It appeared that the DRCNN was over-fitted after the 120th epoch. The best-performing epoch was then manually selected based on minimum cross-entropy loss of the validation set.

### Cascading thresholds

The cascading thresholds technique has been used previously in the literature, in order to boost the sensitivity of a given CNN [35]. Normally, a classifying CNN has a Softmax layer followed by a Classification Output layer as its last layers. The Softmax layer generates a list of probabilities of given input (i.e. fundus photo), to belong to a certain class (i.e. Healthy, Non-referable DR, Referable DR). The Classification Output layer will then choose the class with maximum probability as the outcome of the classifier CNN. Alternatively, to increase the sensitivity of the CNN towards a specific grade (e.g. Referable DR), sub-maximum probabilities of that specific grade could be used.

An example of (~, 0.3, 0.3) cascading thresholds limit is presented here. Following AI’s image analysis, if the output of the Softmax layer for the Referable class reaches the threshold of 0.3, then regardless of the less severe grades probabilities, the image is classified as Referable. If the image is not classified as Referable and if the Softmax layer output of Non-referable DR grade reaches the threshold of 0.3, this image is then assigned to this grade, regardless of the Healthy grade probability. Otherwise, the photos that are not classified as either Referable DR or Non-referable DR, are classified as Healthy. Here, we experimented with the cascading thresholds limits of (~, 0.4, 0.4) and (~, 0.3, 0.3), which are formatted for the corresponding classes: Healthy, Non-referable DR and Referable DR.

### Margin max

To our knowledge, the ‘margin max’ technique has not previously applied in similar studies. In this method, if the top two less sever classes’ probabilities (e.g. Healthy and Non-referable DR) are within a set given threshold, to boost the sensitivity of a certain class (e.g. Healthy) the maximum rule will be ignored. As an example, consider the case of the ‘margin max’ of (0.2) for boosting the sensitivity of the Healthy grade. If the Softmax scores of Healthy, Non-referable and Referable DR were assigned as [0.3–0.45–0.25] respectively, the Healthy grade is chosen although it is not the maximum of three probabilities.

### Microsoft Azure parameters

**Fig 5:**
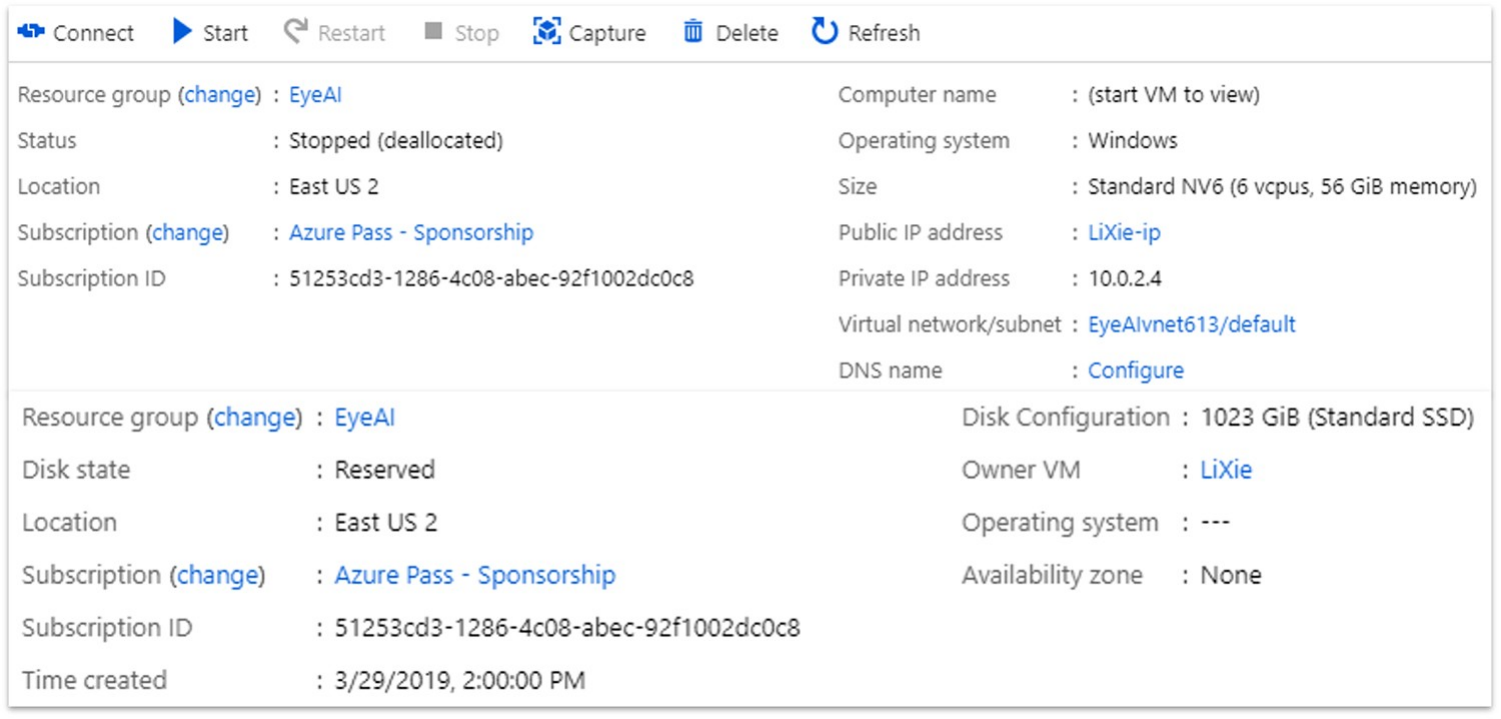
Screenshot of the Microsoft Azure^™^ virtual machine. A Virtual Machine was created on Microsoft Azure East US server. 6 CPUIs were avaialble to us on this Virtual Machine, and it was used for training and validation process.

A standard NV6 Windows instance (6 VCPUs, 56 GB memory) from East US 2 region was selected as the training virtual machine. An additional standard SSD disk of 1023 GB storage space was attached to the training virtual machine [Fig 5]. Resources rented on a cloud platform, such as Microsoft Azure, have a direct impact on the cost of operating an AI as a screening tool. The Azure setup parameters for this project was very modest, as a very expensive but efficient cloud infrastructure (i.e. GPU based Virtual Machines) will become a barrier to its clinical implementation and scalability.

### Definitions used in this paper

Sensitivity, or the true positive rate, is defined as the proportion of people with the disease who will have a positive result:

$$sensitivity=\frac{number\;of\;true\;positives}{number\;of\;true\;positives+number\;of\;false\;negatives}$$

“Specificity”, or the true negative rate, is defined as the proportion of people without the disease who will have a negative result:

$$specificity=\frac{number\;of\;true\;negatives}{number\;of\;true\;negatives+number\;of\;false\;positives}$$

However the utility of the screening tests result will be influenced by the prevalence of the diseased state being screened and as such it is also helpful to calculate the negative and positive predictive values.

The positive predictive value (PPV) is the probability that subjects with a positive screening test is diseased, defined by:

$$PPV=\frac{number\;of\;true\;positives}{number\;of\;true\;positives+number\;of\;false\;positives}$$

The negative predictive value (NPV) is the probability that subjects with a negative screening test is disease free, defined by:

$$NPV=\frac{number\;of\;true\;negatives}{number\;of\;true\;negatives+number\;of\;false\;negatives}$$

In a disease like sight-threatening DR, where the prevalence of the diseased state is low (<5%), a tool which drives the false negative rate to near zero will therefore generate not only a high sensitivity, but also a very high negative predictive value of a negative test result.

F1 score is a measurement of classification accuracy that considers both positive predictive value and sensitivity and is defined by:

$$F1\;score=2*\frac{PPV*sensitivity}{PPV+sensitivity}$$

## Results

### Generate the curated testing set

The “Quality Assessment” CNN reached 99% accuracy and the validation loss of lower than 0.05. This CNN model was then used to create a ‘curated’ test set from the Kaggle EyePACS dataset. The ‘curated’ test set included 6,900 images from the original 13,305 ‘un-curated’ set (i.e. a 47% rejection rate).

### Un-curated testing set versus curated testing set

The DRCNN was trained and validated using the Microsoft Azure^™^ cloud platform. This was done twice, once for the binary DR grading classification Healthy vs Diseased, and once for the DR grading classification Healthy, Non-referable DR, and Referable DR. The cross-entropy and accuracy were tracked and recorded throughout the training and validation process. The training progress was monitored for 140 epochs and the best set of weights that resulted in minimal validation loss was picked and set for the proceeding CNN performance assessment.

While, the DRCNN was trained and validated using ‘un-curated’ data, it was tested separately using unseen ‘curated’ and ‘un-curated’ data. One would assume that using ‘curated’ (i.e. higher quality) data for the CNN test would improve the performance of the model. Here and for the first time, we wanted to assess this hypothesis [Tables 3&4].

**Table 3:** Performance of the DRCNN based on three grades (healthy, non-referable, referable).

	‘un-curated’ test set	‘curated’ test set
**accuracy**	0.8681	0.8884
**Sensitivity Healthy**	0.9665	0.9726
**Sensitivity Non-referable DR**	0.5855	0.6317
**Sensitivity Referable DR**	0.6093	0.6087
**Specificity Healthy**	0.9005	0.9127
**Specificity Non-referable DR**	0.7770	0.8163
**Specificity Referable DR**	0.6232	0.6300
**F1 Score Healthy**	0.9323	0.9417
**F1 Score Non-referable DR**	0.6678	0.7122
**F1 Score Referable DR**	0.6162	0.6192
**PPV Healthy**	0.9005	0.9127
**PPV Non-referable DR**	0.7770	0.8163
**PPV Referable DR**	0.6232	0.6300
**NPV Healthy**	0.8801	0.8943
**NPV Non-referable DR**	0.8934	0.9051
**NPV Referable DR**	0.9814	0.9879

**Table 4:** Performance of the DRCNN based on two grades (healthy, diseased).

	‘un-curated’ test set	‘curated’ test set
**accuracy**	0.8963	0.9091
**Specificity Healthy**	90.05	91.27
**Specificity Diseased**	88.01	89.43
**Sensitivity Healthy**	96.65	97.26
**Sensitivity Diseased**	69.75	71.32
**F1 Score Healthy**	0.9323	0.9417
**F1 Score Diseased**	0.7783	0.7935
**PPV Healthy**	0.9005	0.9127
**PPV Diseased**	0.8801	0.8943
**NPV Healthy**	0.8801	0.8943
**NPV Diseased**	0.9005	0.9127

Interestingly, the DRCNN prediction performance improved only marginally for the ‘curated’ test sets, compared to the ‘un-curated’ set.

### Sensitivity uplift

Several implementations of ‘cascading thresholds’ and ‘margin max’ techniques were then used to boost the sensitivity of the DRCNN, using the ‘curated’ and ‘un-curated’ test sets, for both two and three grading level schemes.

It appeared that Cascading Thresholds (~, 0.3, 0.3) and Margin Max (0.4) were the most effective techniques for sensitivity boosting. We then investigated the effects of these techniques to boost the sensitivity of CNN towards either the Healthy or most Diseased grade [Tables 5–8].

**Table 5:** Sensitivity boost of the ‘curated’ dataset with three labels, for healthy and diseased categories.

	Margin Max (0.4) Boosting Healthy	Original	Cascading Thresholds (~, 0.3, 0.3) Boosting Diseased
accuracy	0.8827	0.8884	0.8721
Sensitivity Healthy	0.9852	0.9726	0.9364
Sensitivity Non-referable DR	0.5755	0.6317	0.6676
Sensitivity Referable DR	0.5072	0.6087	0.7199
Specificity Healthy	0.8957	0.9127	0.9291
Specificity Non-referable DR	0.8374	0.8163	0.7245
Specificity Referable DR	0.6954	0.63	0.5284
F1 Score Healthy	0.9383	0.9417	0.9327
F1 Score Non-referable DR	0.6822	0.7122	0.6949
F1 Score Referable DR	0.5866	0.6192	0.6094
PPV Healthy	0.8957	0.9127	0.9291
PPV Non-referable DR	0.8374	0.8163	0.7245
PPV Referable DR	0.6954	0.6300	0.5284
NPV Healthy	0.9340	0.8943	0.7991
NPV Non-referable DR	0.8930	0.9051	0.9109
NPV Referable DR	0.9848	0.9879	0.9912

**Table 6:** Sensitivity boost of the ‘un-curated’ dataset with three labels, for healthy and diseased categories.

	Margin Max (0.4) Boosting Healthy	Original	Margin Max (0.4) Boosting Diseased
accuracy	0.8680	0.8681	0.8444
Sensitivity Healthy	0.9831	0.9665	0.9154
Sensitivity Non-referable DR	0.5503	0.5855	0.6203
Sensitivity Referable DR	0.5026	0.6093	0.7522
Specificity Healthy	0.8840	0.9005	0.923
Specificity Non-referable DR	0.8004	0.777	0.6707
Specificity Referable DR	0.7624	0.6232	0.5052
F1 Score Healthy	0.9309	0.9323	0.9192
F1 Score Non-referable DR	0.6522	0.6678	0.6445
F1 Score Referable DR	0.6058	0.6162	0.6044
PPV Healthy	0.8840	0.9005	0.923
PPV Non-referable DR	0.8004	0.7770	0.6707
PPV Referable DR	0.7624	0.6232	0.5052
NPV Healthy	0.9297	0.8801	0.7659
NPV Non-referable DR	0.8862	0.8934	0.8978
NPV Referable DR	0.9767	0.9814	0.9879

**Table 7:** Sensitivity boost of the ‘curated’ dataset with two labels, for healthy and diseased categories.

	Margin Max (0.4) Boosting Healthy	Original	Margin Max (0.4) Boosting Diseased
accuracy	0.9022	0.9091	0.8968
Sensitivity Healthy	0.9852	0.9726	0.9343
Sensitivity Diseased	0.6467	0.7132	0.7815
Specificity Healthy	0.8957	0.9127	0.9294
Specificity Diseased	0.9340	0.8943	0.7942
F1 Score Healthy	0.9383	0.9417	0.9319
F1 Score Diseased	0.7642	0.7935	0.7878
PPV Healthy	0.8957	0.9127	0.9294
PPV Diseased	0.9340	0.8943	0.7942
NPV Healthy	0.9340	0.8943	0.7942
NPV Diseased	0.8957	0.9127	0.9294

**Table 8:** Sensitivity boost of the ‘un-curated’ dataset with two labels, for healthy and diseased categories.

	Margin Max (0.4) Boosting Healthy	Original	Margin Max (0.4) Boosting Diseased
accuracy	0.8921	0.8963	0.8810
Sensitivity Healthy	0.9831	0.9665	0.9154
Sensitivity Diseased	0.6346	0.6975	0.7836
Specificity Healthy	0.8840	0.9005	0.923
Specificity Diseased	0.9297	0.8801	0.7659
F1 Score Healthy	0.9309	0.9323	0.9192
F1 Score Diseased	0.7543	0.7783	0.7746
NPV Healthy	0.9297	0.8801	0.7659
NPV Diseased	0.884	0.9005	0.923
PPV Healthy	0.8840	0.9005	0.9230
PPV Diseased	0.9297	0.8801	0.7659

It appeared that boosting the sensitivity using both ‘cascading thresholds’ and ‘margin max’ had a similar effect for ‘curated’ and ‘un-curated’ datasets. Also, it seemed that uplifting the sensitivity of the Healthy grade, also enhanced the specificity of the Diseased state, and vice versa.

Here we have shown [Tables 7 & 8] that by adjusting the post processing of the outcome of a CNN, we have outperformed the previously published best performance of Kaggle EyePACS, which was later failed to be replicated [37].

There have been several studies that have used the EyePACS dataset for training a DR AI [28, 44–47]. However, these studies are not directly comparable to the DRCNN presented here, as each study uses different (sometimes private) training sets, validation sets, DR grading schemes and performance reporting metrics. Regardless, a comparison of the performance of these models against the un-boosted and boosted DRCNN is provided here [Table 9]. It can be seen that while our DRCNN is not the best performing neural network (although different studies are not directly comparable), different performance aspects of it (sensitivity to healthy or diseased, etc.) would outperform other published CNNs, depending on the boosting strategy. The confusion matrix of each study mentioned here is presented as a S1 Data.

**Table 9:** Comparison of un-boosted and boosted DRCNN with previous studies, which used the EyePACS dataset. The highlighted cells show the ‘performance gains’ due to different boosting strategy.

		Ghosh et al [44]	Pratt et al [28]	Kwasigroch et al [45]	Raju et al [46]	Qummar et al #1 [47]	Qummar et al #2 [47]	DRCNN	DRCNN boosted for Healthy	DRCNN boosted for Diseased
**Healthy**	**Sensitivity**	95.04	95.05	50.5	92.29	97.35	97.26	97.26	98.52	93.64
**Non-Referable**		55.86	18.09	60.25	62.45	34.87	77.04	63.17	57.55	66.76
**Referable**		80	31.56	84.75	81.65	62.11	75.09	60.87	50.72	71.99
**Healthy**	**F1 score**	91.5	85.09	52.47	92.62	90.32	80.66	94.17	93.83	93.27
**Non-Referable**		65.49	26.14	60.78	65.23	46.70	75.64	71.22	68.22	69.49
**Referable**		74.51	37.67	82.48	64.7	64.11	85.33	61.92	58.66	60.94
**Healthy**	**Specificity**	67.19	29.99	89.5	80.28	49.66	88.62	71.32	64.67	77.97
**Non-Referable**		95.86	94.02	74.67	91.95	95.91	83.5	96.11	96.94	93.05
**Referable**		97.80	98.3	86.17	96.65	98.49	99.36	98.89	99.31	98.00
**Healthy**	**NPV**	83.99	69.44	87.85	78.71	87.18	99.2	89.43	93.40	79.91
**Non-Referable**		88.54	79.55	73.81	89.84	83.9	85.45	90.51	89.30	91.09
**Referable**		98.72	96.82	89.45	99.11	98.19	85.07	98.79	98.48	99.12
	**Accuracy**	85.55	74.66	68.1	85.33	81.98	80.40	88.84	88.27	87.21
**Healthy**	**Sensitivity**	95.04	95.05	50.5	92.29	97.35	97.26	97.26	98.52	96.05
**Referable**		67.19	29.99	89.5	80.28	49.56	89.03	71.23	64.67	73.99
**Healthy**	**F1 score**	91.5	85.9	52.47	96.62	90.32	80.66	94.17	93.83	93.94
**Referable**		74.66	41.88	88.67	79.49	63.18	93.85	79.35	76.42	79.94
**Healthy**	**Specificity**	67.19	29.99	89.5	80.28	49.56	89.03	71.32	64.67	73.99
**Referable**		95.04	95.05	50.5	92.29	97.35	97.26	97.26	98.52	96.05
**Healthy**	**NPV**	83.99	69.44	87.85	78.71	87.13	99.24	89.43	93.40	85.87
**Referable**		88.22	78.35	54.59	92.95	84.23	68.90	91.27	89.57	91.92
	**Accuracy**	87.27	77.3	81.7	89.14	84.66	90.67	90.9	90.22	90.64

## Discussion

Diabetic retinopathy (DR) is the most common microvascular complication of diabetes and is the leading cause of blindness among the working-age population [48]. Whilst the risk of sight loss from DR can be reduced through good glycaemic management [49], if sight-threatening DR develops, timely intervention with laser photocoagulation or injections of anti-vascular endothelial growth factor is required [50, 51]. Thus, if the risk of sight loss is to be reduced, individuals with diabetes should have their eyes screened regularly to facilitate the detection of DR, before vision loss occurs [52]. Unfortunately, in many regions including New Zealand, the attendance at DR screening falls below the recommended rates [53–55], and this is particularly true for those who live in remote areas and those of lower socioeconomic status [56–58]. There remain then significant challenges to ensure that delivery of these services is equitable and that all patients at risk are being screened regularly. Currently, the existing model of DR screening is resource intensive, requiring a team of trained clinicians to read the photographs. They also have high capital setup costs, related to the retinal cameras required to take the photographs, and require an efficient administrative IT support system to support and run them. As a result, the existing DR programs are relatively inflexible and are not easily scalable. All these issues are more acute in the developing world, which lack both capital funds and trained healthcare professionals [59]. Incorporating AI to accurately grade the retinal images for DR would offer many benefits to DR screening programs; reducing their reliance on trained clinicians to read photographs, enabling point of contact diagnosis whilst reducing the need for complex IT support systems. Research into AI design and its development for DR screening has progressed significantly in recent years, and this field has enjoyed a good deal of attention of late [60–62]. However, for all the excitement very little of this work has progressed to a clinically useful tool which provides a real-world AI-solution for DR screening programs and this is due largely to the challenges of the research-driven AI to generalize to a real-world setup. Whilst there are many reasons for such a lack of generalisation, the principal ones are the use of small and ‘curated’ datasets and an emphasis on overall accuracy, rather than sensitivity of the developed AI. The AI’s reliance on powerful computers that are not available in most clinical environments has been an additional contributory factor.

Traditionally, several metrics have been used to describe the performance of a DRCNN including, but not limited to accuracy, sensitivity, specificity, precision, negative and positive predictive values. However traditional screening is a binary exercise, categorising patients into those at low risk of having disease from those at high risk of having disease. As such, there is trade-off between the need for a high sensitivity with an acceptable specificity. Traditional diabetic eye screening programs have therefore mandated a minimum sensitivity of >85% and specificity of >80% for detecting sight-threatening diabetic retinopathy as there is a personal and financial cost associated with unnecessary referrals to eye clinics [63]. Whilst it is appropriate that a screening *program* has to strike the correct balance between these parameters, we envisage that in many situations, a classifier DRCNN will not be the sole arbitrator for grading diabetic retinopathy. It is therefore appropriate to consider the role that a classifier DRCNN could play in a diabetic eye screening program as currently there has been very little discussion of this subject.

In this study, we report the results of two techniques, ‘cascading thresholds’ and ‘margin max’ to assess how they could be used to drive up the sensitivity, the negative predictive value, or the specificity and the positive predictive value in bespoke DRCNN’s depending on the applications mode. In doing so, we boosted the AI’s sensitivity to detect Healthy cases to more than 98%, while also improving the specificity of the other more severe classes. These techniques also boosted the AI’s sensitivity of referable disease classes to near 80%.

Using the techniques described in this paper, it then becomes possible to develop classifier DRCNNs, tailored to the specific requirements of the DR program they have been commissioned to support [63]. The United Kingdom national Ophthalmology database study revealed that of the 48,450 eyes with structured assessment data at the time of their last record, 11,356 (23.8%) eyes had no DR and 21,986 (46.0%) had “non-referable” DR [64]. Thus a sensitivity boosted classifier like the one described here, manipulated to detect eyes with referable DR with a very high sensitivity (>95%) and ultra-high negative predictive value (>99.5%), could be embedded into an existing eye screening program rapidly and safely triaging eyes with referable disease from eyes which do not. Such a DRCNN would reduce the number of images sent to the human grading team for review by between 70–80%, leading to immediate and significant cost savings for the program.

In the context of a rural or developing-world setting, the ability to identify those patients with no disease from those with any disease may be desirable as it is well-recognised that the development of *any* DR, no matter how “mild” is a significant event [65]. Thus its detection could be used to target valuable and scarce health care resources more effectively to those individuals at highest risk to ensure that their diabetes and blood pressure control are reviewed. In effect, even if no other CNN approach was then used, the relatively simple cloud-based DRCNN we described here would help identify those patients at increased risk of either advanced disease or disease progression, and who therefore merit review of their systemic disease. Moreover, using the techniques described here, more sophisticated classifier CNNs could also be developed, ones that are manipulated to detect disease with a very high sensitivity. It is even conceivable that different classifier CNNs could then be run concurrently within diabetic eye screening programs to sequentially grade differing levels of disease with high sensitivity, ultimately leaving the human grading team with a relatively small number of images to review for adjudication and quality assurance.

Arguably, one of the biggest challenges that faces all AI-based “diagnostic” systems is the issue of public trust. Whilst it is accepted that in a traditional screening program with a sensitivity of 90%, 1 in 10 patients will be informed that are healthy when in actual fact they have disease, well-publicised failures of AI systems suggest that the public would not accept such failure rates from a “computer”. In this context, the negative predictive value is arguably more important than traditional sensitivity and specificity. Whilst the relatively simple CNN described in this paper lacks the required sensitivity to be the sole arbitrator for identifying referable disease in a structured screening program, the fact that the methods we describe boosted both the sensitivity of the DRCNN to detect disease by over 10%, and thus the negative predictive value to near 100%, is noteworthy. We therefore believe that the techniques we describe here will prove to be valuable tools for those looking to build bespoke CNN’s in the future.

In this research, we have endeavoured to address those issues that hinder the clinical translation of an in-house bespoke AI for DR screening. Our DRCNN was developed and tested using real-world ‘un-curated’ data. Here we demonstrated that our DRCNN is ‘robust’, as its performance is not critically affected by the quality of the input data. Furthermore, this process of data management, model training and validation was performed using Microsoft’s Azure^™^ cloud platform. In doing so, we have demonstrated that one can build AI that is constantly re-trainable and scalable through cloud computing platforms. Although few DRCNN’s are accessible online, to our knowledge this is the first time that an AI has been fully implemented and re-trainable through a cloud platform. Hence, provided there is internet access, our DRCNN is capable of reaching remote and rural places; areas traditionally not well served by existing DR screening services.

In conclusion, we have demonstrated how existing machine learning techniques can be used to boost the sensitivity, and hence negative predictive value, and specificity of a DRCNN classifier. We have also demonstrated how even a relatively simple classifier CNN, one that is capable of running on a cloud-based provider, can be utilised to support both existing DR screening programs and the development of new programs serving rural and hard to reach communities. Further work is required to both develop classifiers that can detect sight-threatening DR with a very high sensitivity, and evaluate how a battery of DRCNN’s each with differing specifications and roles, may be used concurrently to develop a real-world capable, fully automated DR screening program.

## Supporting information

S1 Data (DOCX)
