# Supplementary material for: Towards implementation of AI in New Zealand national diabetic screening program: Cloud-based, robust, and bespoke
Source: PLoS One. 2020 Apr 10;15(4):e0225015. doi: 10.1371/journal.pone.0225015 (PMC7147747; doi:10.1371/journal.pone.0225015)
Supplement: S1 Data — (DOCX) [file pone.0225015.s001.docx]

**Supplementary data**

Ghosh et al. [44]

|  | Healthy | Non-referable | Referable |
| --- | --- | --- | --- |
| Healthy | 2186 | 76 | 38 |
| Non-referable | 281 | 391 | 28 |
| Referable | 11 | 27 | 152 |

|  | Healthy | Referable |
| --- | --- | --- |
| Healthy | 2186 | 114 |
| Referable | 292 | 598 |

Pratt et al. [28]

|  | Healthy | Non-referable | Referable |
| --- | --- | --- | --- |
| Healthy | 3456 | 145 | 35 |
| Non-referable | 887 | 206 | 46 |
| Referable | 68 | 86 | 71 |

|  | Healthy | Referable |
| --- | --- | --- |
| Healthy | 3456 | 180 |
| Referable | 955 | 409 |

Kwasigroch et al. [45]

|  | Healthy | Non-referable | Referable |
| --- | --- | --- | --- |
| Healthy | 101 | 95 | 4 |
| Non-referable | 80 | 241 | 79 |
| Referable | 4 | 57 | 339 |

|  | Healthy | Referable |
| --- | --- | --- |
| Healthy | 101 | 99 |
| Referable | 84 | 716 |

Raju et al. [46]

|  | Healthy | Non-referable | Referable |
| --- | --- | --- | --- |
| Healthy | 36484 | 2960 | 89 |
| Non-referable | 2741 | 7259 | 1623 |
| Referable | 28 | 416 | 1976 |

|  | Healthy | Referable |
| --- | --- | --- |
| Healthy | 36484 | 3049 |
| Referable | 2769 | 11274 |

Qummar et al. [47]

|  | Healthy | Non-referable | Referable |
| --- | --- | --- | --- |
| Healthy | 4010 | 100 | 9 |
| Non-referable | 733 | 431 | 72 |
| Referable | 18 | 79 | 159 |

|  | Healthy | Referable |
| --- | --- | --- |
| Healthy | 4010 | 109 |
| Referable | 751 | 738 |

Qummar #2 et al. [40]

|  | Healthy | Non-referable | Referable |
| --- | --- | --- | --- |
| Healthy | 4006 | 103 | 10 |
| Non-referable | 1692 | 5895 | 65 |
| Referable | 116 | 1936 | 6186 |

|  | Healthy | Referable |
| --- | --- | --- |
| Healthy | 4006 | 113 |
| Referable | 1808 | 14668 |

DRCNN

|  | Healthy | Non-referable | Referable |
| --- | --- | --- | --- |
| Healthy | 5047 | 136 | 6 |
| Non-referable | 476 | 933 | 68 |
| Referable | 7 | 74 | 126 |

|  | Healthy | Referable |
| --- | --- | --- |
| Healthy | 5047 | 142 |
| Referable | 483 | 1201 |

DRCNN boosted for Healthy

|  | Healthy | Non-referable | Referable |
| --- | --- | --- | --- |
| Healthy | 5112 | 72 | 5 |
| Non-referable | 586 | 850 | 41 |
| Referable | 9 | 93 | 105 |

|  | Healthy | Referable |
| --- | --- | --- |
| Healthy | 5112 | 77 |
| Referable | 595 | 1089 |

DRCNN boosted for Diseased

|  | Healthy | Non-referable | Referable |
| --- | --- | --- | --- |
| Healthy | 4859 | 323 | 7 |
| Non-referable | 365 | 986 | 126 |
| Referable | 6 | 52 | 149 |

|  | Healthy | Referable |
| --- | --- | --- |
| Healthy | 4984 | 205 |
| Referable | 438 | 1246 |
